# Neurodevelopmental assessment of early treated children with phenylketonuria: insights from Griffith III scales

**DOI:** 10.1007/s00431-025-06384-2

**Published:** 2025-08-15

**Authors:** Zahraa Abdelmoneim, Heba Eltaher, Mohamed Abdelghafar Hussein, Menna E. Hashish

**Affiliations:** 1 Departement of Pediatrics, Genetics and Metabolic Unit, Faculty of Medicine, Mansoura University, Mansoura, Egypt; 2 Department of Pediatrics, Faculty of Medicine, Mansoura University, Mansoura, Egypt; 3 Department of Pediatrics, Faculty of Medicine, Kafrelsheikh University, Kafrelsheikh, Egypt

**Keywords:** Phenylketonuria, Griffiths III developmental scales, Neurodevelopmental profile

## Abstract

Neonatal screening of phenylketonuria (PKU) and early treatment are fundamental to prevent mental retardation in children. Unfortunately, it has been observed that some neurological sequelae may still be exhibited despite these preventive strategies. Assessment of the neurodevelopment of early treated children may aid in understanding the devastating effect of PKU on the developing brain, so this study aimed to investigate the neurodevelopmental outcome of early-treated children with PKU using Griffiths-III developmental scales. We conducted an observational single-center case-control study on a total of 60 children.  We compared the neurodevelopmental profile of two groups of children (PKU group = 30 and healthy control group = 30) using Griffiths-III developmental scales. Also PKU children were divided into two subgroups according to their phenylalanine level: controlled and uncontrolled. There were significant decreases in the mean of developmental quotients (DQs) of Griffiths-III subscales A, B, C, D, and general development of PKU group. While there was insignificant difference in the DQ of subscale E (gross motor) among the two groups, there was a significant difference between the two PKU subgroups regarding the developmental quotient of subscales A, B, C, E, and general development. Also, there was a statistically significant correlation between phenylalanine (Phe) levels and the mean of DQs of all Griffiths-III subscales.

*Conclusion*: Early- treated PKU children are at risk of poor neurodevelopmental outcome even if their gross motor function is normal and this defect is negatively correlated with phenylalanine levels.

**Table:** 

**What is Known:** • *PKU is an inborn error of metabolism that causes mental retardation if not treated early*. • *Neonatal screening and early treatment prevent mental retardation*.	
**What is New:** • *Despite neonatal screening and early treatment, PKU children still exhibit mental function impairment*. • *The Griffiths III developmental scales is the first time to be used to assess mental functions in PKU children*.

**What is Known:**

• *PKU is an inborn error of metabolism that causes mental retardation if not treated early*.

• *Neonatal screening and early treatment prevent mental retardation*.

**What is New:**

• *Despite neonatal screening and early treatment, PKU children still exhibit mental function impairment*.

• *The Griffiths III developmental scales is the first time to be used to assess mental functions in PKU children*.

## Introduction

Phenylketonuria (PKU, MIM #261600) is an autosomal recessive disease that impacts the conversion of amino acid Phe into tyrosine, resulting in significant consequences for brain health [1]. PKU is the most common inborn error of amino acid metabolism in Egypt with a relatively higher incidence of 1/3000 (0.03%), which means that about 333 neonates are affected with PKU every year as 1 million babies are born yearly [2]. In untreated individuals, the disease is characterized by irreversible intellectual disability, seizures, behavioral abnormalities, microcephaly, and dermatological issues all resulting from hyperphenylalaninemia (HPA). Other clinical manifestations include gait abnormalities, altered sitting posture, and abnormal stance. The body and urine may emit a “mousy” odor due to elevated levels of phenylacetic acid. In the absence of dietary restrictions, cognitive impairment worsens during myelination in early childhood [3].

Since the establishment of the national neonatal PKU screening initiative in Egypt in collaboration with the Ministry of Health (MOH) in 2015, infants diagnosed with PKU have been placed on a Phe-restricted diet, which reduces blood Phe levels and prevents mental disability. It is now advised that individuals with PKU maintain this dietary regimen throughout their lifetime [4]. Life-long treatment is recommended to maintain normal phenylalanine level (120–360 μmol/L) as bad metabolic control and greater phenylalanine variations are associated with worsening of the executive functions [5]. However, it has been observed that patients who receive continuous dietary intervention following diagnosis through newborn screening may still exhibit some neurological sequelae, although these are significantly less severe than those seen in untreated patients. One cognitive domain particularly affected is executive and non-executive mental (frontal lobe) function. Mild impairment in this area is evident even in early-treated PKU (ETPKU) patients [6].

Variations in Phe can cause brain damage, resulting in cognitive impairment, hyperactivity, and deficiencies in executive functions and social cognition [7]. A 100 mmol/L increase in phenylalanine levels is associated with a 1.3 to 6 point drop in intelligence quotient [8]. Adherence to a healthy diet is crucial for both metabolic stability and cognitive development.

Although dietary treatment has been demonstrated to improve cognitive and behavioral results, early intervention may still result in neuropsychological dysfunctions. Long-term fluctuations in Phe levels appear to have a higher impact on the executive functions [9].

Improved management of PKU can be achieved through the broader use of current pharmacological treatments as well as the introduction of new therapies [10]. However, it also relies on a deeper understanding of how the cognitive impairments observed in these patients are linked to variations in Phe levels, so cognitive assessment is very vital [11]. The Griffiths III Scales of Child Development are widely used for neurodevelopmental assessment, evaluating both executive and non-executive mental functions, and have demonstrated ongoing validity and accuracy globally. The Griffiths III scales offer a comprehensive measure of child development from 1 day to 5 years and 11 months (71 months) [12].

Enhanced management of PKU can be attained through expanding existing treatments and implementing novel therapies. However, it also relies on a deeper understanding of how the cognitive impairments observed in these patients are linked to variations in Phe levels, so cognitive assessment is very vital [13]. In the current study we aimed to assess the neurodevelopmental outcome of ETPKU children using Griffiths-III developmental scales.

## Subjects and methods

The study was an observational case–control study done from March 2024 to March 2025. This study was approved by the Ethics Committee of the Mansoura Faculty of Medicine—Institutional Research Board (Code number: R.24.01.2483). All children aged below 6 years—as Griffith’s assessment scale is not applicable above 6 years old—diagnosed with PKU through newborn screening (NBS) in the first week of life and received the prescribed phe-free medical formula immediately after diagnosis in the neonatal period, were included in the study. Those PKU children who were diagnosed late after CNS insult; PKU children who did not receive a Phe-restricted diet at all; refused treatment; stopped follow-up; patients with any systemic disease affecting cognitive function, brain anomalies, intellectual disabilities, or other genetic or metabolic disorders were excluded. Also, BH4 deficiency children were excluded as they are mentally retarded.

Sixty children were included in our study and were categorized into two groups: 30 ETPKU children (diagnosed through NBS) and confirmed by a second blood sample through the NBS program) were recruited from the metabolic clinic at Mansoura University Children Hospital and 30 healthy children as a control group.

The newborn screening used Whatman 903 filter paper to detect and dry blood from a heel stick for screening. The sampling period was between the third and seventh days of life of all newborns. Dried blood spots were preprocessed according to the NeoBaseTM non-derivatized MS/MS kit instructions before being analyzed with the TQD MS/MS system and NeoBaseTM non-derivatized system. The analyses include Phe, tyrosine, and acylcarnitines. Positive cases underwent plasma amino acid analysis using chromatography to detect Phe levels [14].

When an infant has a positive newborn screen for PKU and a diagnosis is confirmed by elevated Phe more than 360 μmol/L so treatment is required, the first step is to lower the blood phenylalanine content to a treatment range of 120–360 μmol/L (2–6 mg/dL). This is achieved by washout period by stoppage of breast or artificial milk and giving only the special Phe-free medical formula for 24 h if initial Phe is 360–600 μmol/L, for 48 h if initial Phe is 600–1200 μmol/L, for 72 h if initial Phe is 1200–2400 μmol/L, and 96 h if initial Phe is > 2400 μmol/L [9].

Once the blood phenylalanine concentration trends down toward the treatment range, a source of intact protein is added to provide the infant’s phenylalanine needs from breast or artificial ordinary formula. Chronic dietary management for PKU involves giving a diet free of phenylalanine as a source of protein requirement (from the Phe-free medical formula) and giving the calculated phenylalanine requirement from a low-protein diet to prevent phenylalanine toxicity or deficiency. This can be achieved by natural protein restriction, Phe-free L-amino acid supplements, and low-protein food [8].

We adjust the amount of medical formula and the low-protein diet according to the Phe level, nutritional status, and neurological assessment at every visit. We measure Phe level and tyrosine by tandem mass. Also Phe/tyrosine ratio is determined and should not be elevated.

The frequency of follow-up Phe measurement varies based on the child's age as follows: weekly in the first 2 months, every 2 weeks from 2 to 6 months of age, monthly from 6 to 12 months of age, every 3 months from 1 to 8 years of age, and every 6 months for children over 8 years of age.

Our cases were classified according to the initial serum Phe level into classic PKU (serum Phe plasma concentrations in an untreated, newly diagnosed newborn exceed 20 mg/dL (1200 μmol/L), moderate PKU (phe concentrations 900 to 1200 μmol/L), and mild PKU (phenylalanine concentrations 600 to 900 μmol/L) [15]. The mean of their serum Phe level follow-up in the previous year and in the last 3 months before the neurodevelopmental assessment was also recorded.

### Sample size

The sample size calculation was done by G*Power 3.1.9.2. (Universitat Kiel, Germany). According to a previous study, the mean ± SD of the executive function assessment in PKU patients was 1.91 ± 0.86, and in healthy controls was 2.64 ± 0.98 [16]. The sample size was based on the following considerations: 0.792 effect size, 95% confidence level, 80% power of the study, and group ratio 1:1. Sample size is 27 children in each group, three cases were added in each group to overcome dropout, so we aimed to recruit about 30 children in each group.

### Neurodevelopmental assessment

Griffiths III developmental scales were used by the trained examiner, unaware of the clinical history and the laboratory results for the studied subjects. The administration time ranged from approximately 50 to 90 min, depending on the child’s age. The child underwent assessments in five subscales: foundation of learning (subscale A), language and communication (subscale B), eye and hand coordination (subscale C), personal-social-emotional (subscale D), gross motor (subscale E), and general development. The raw score of each subscale was converted into percentile ranks (1–99%), DQ (range 50–150) and developmental age equivalents (DA) in months through part III norms book of Griffiths-III [12].

### Statistical analysis

Data analysis was performed by SPSS software, version 24 (SPSS Inc., PASW statistics for Windows version 24. Chicago: SPSS Inc.). Qualitative data are expressed as frequencies and percentages. Quantitative data were described using median (minimum and maximum) (interquartile range) for non-normally distributed data and mean ± standard deviation for normally distributed data after testing normality using the Kolmogorov–Smirnov test. The significance of the results obtained was judged at the 0.05 level. The Fisher exact test was used to compare qualitative data between groups as appropriate. Mann–Whitney *U* was used to compare between two studied groups for non-normally distributed data. Student *t*-test was used to compare two independent groups for normally distributed data. Pearson correlation coefficient was used to measure the linear correlation between follow-up Phe level and Griffiths-III developmental scales.

## Results

Table 1 shows the demographic characteristics among PKU and control groups. Median ages were 48.5 months and 40 months for PKU and healthy children, respectively. Positive family history and consanguinity was in 26.7% and 73.3% of PKU cases, respectively. A total of 63.3% were from rural areas while 36.7% were from urban areas. All PKU cases were diagnosed in the first week of life through the NBS program and started the Phe-free medical formula immediately after confirming the diagnosis. Furthermore, the diseased children were classified according to their initial Phe levels into 13 cases as classic, 6 cases as moderate, and 11 cases as mild PKU.

The median of initial Phe level at diagnosis was 970 μmol/L, the mean of Phe level in the previous year before assessment was 845.33 ± 508 μmol/L, and in the previous 3 months was 845.7 ± 516 μmol/L.

**Table 1:** Demographic data of both groups

	PKU *N* = 30	Control *N* = 30	*P* value
Age in months (median (min–max))	48.5 (14–72)	44 (12–72)	0.228*
Sex (M/F)	12/18	16/14	0.98**
Family history No (%)	8 (26.7%)		
Consanguinity No (%)	22 (73.3%)		
Residency No (%)	Urban 11 (36.7%)		
	Rural 19 (63.3%)		
PKU phenotypes among cases	Classic 13		
	Moderate 6		
	Mild 11		
Initial Phe (μmol/L) Median (IQR)	970 (695–1500)		
Follow-up Phe in the previous year (μmol/L) Mean ± SD	845.33 ± 508		
Follow-up Phe in the previous 3 months (μmol/L) Mean ± SD	845.7 ± 516		

*Mann–Whitney U test

**FE, Fisher exact test

*PKU* phenylketonuria, *Phe* phenylalanine, *IQR* interquartile range

Table 2 shows the Griffith-III developmental scales among the studied groups. There was a significant decrease in the mean of DQ of subscales A, B, C, D, and general development in the PKU group compared to the healthy group, while the difference in DQ of subscale E among both groups was insignificant.

**Table 2:** Griffiths-III developmental scales among the studied groups

	PKU (30)	Control (30)	*P* value
	Median (min–max)(mean ± SD)	Median (min–max)(mean ± SD)	
subscaleA_percentile	9 (0–83)	79 (16–98)	< 0.001**
subscaleA_DQ (mean ± SD)	79.87 ± 18.83	113.33 ± 11.57	< 0.001*
subscaleB_percentile	8 (1–76)	73.5 (20–92)	< 0.001**
subscaleB_DQ	77.5 (63–111)	110 (87–122)	< 0.001**
subscaleC_percentile	29 (1–85)	71.5 (18–94)	< 0.001**
subscaleC_DQ (mean ± SD)	89.2 ± 18.07	108.6 ± 9.9	< 0.001*
subscaleD_percentile	54 (0–97)	78.5 (37–94)	< 0.004**
subscaleD_DQ (mean ± SD)	97.6 ± 19.13	111.3 ± 8.1	0.001*
subscaleE_percentile (mean ± SD)	56.6 ± 27.29	67.67 ± 20.57	0.081*
subscaleE_DQ (mean ± SD)	103.8 ± 14.05	109.17 ± 10.6	0.102*
general_percentile	24 (0–95)	79 (30–92)	< 0.001**
general_DQ (mean ± SD)	87.7 ± 20.9	111.6 ± 8.3	< 0.001*

*Independent *t*-test

**Mann–Whitney *U* test

The bold *P*-value reflects a significant difference  *PKU* phenylketonuria, *SD* standard deviation, *IQR* interquartile range, *DQ*, developmentalquotient

We divided PKU children into two subgroups according to their phenylalanine level: controlled (phenylalanine less than or equal to 360 μmol/L) and uncontrolled (phenylalanine more than 360 μmol/L) according to PKU European Guidelines [8].

Table 3 shows a significant difference between the two subgroups regarding the developmental quotient of subscales A, B, C, E, and general development.

**Table 3:** Griffiths-III developmental scales among the controlled and uncontrolled PKU children

	SubscaleA_DQ	Subscale B_DQ	Subscale C_DQ	Subscale D_DQ	Subscale E_DQ	Subscale general_DQ
Controlled (***N*** = 5)	95.8 ± 14.58	105 (83–111)	105.4 ± 14.3	111 ± 14.7	118.4 ± 13.5	108.8 ± 17
Uncontrolled (***N*** = 25)	76.68 ± 18.16	77 (63–110)	86.04 ± 17.2	94.96 ± 19.01	100.9 ± 12.4	83.4 ± 19.1
***P* value**	0.036*	0.004**	0.026*	0.087*	0.009*	0.011*

*Independent *t*-test

**Mann–Whitney *U* test

The bold *P*-value reflects a significant difference

Table 4 shows the correlation between Phe level follow-up in the last 1 year and the last 3 months and DQ of Griffiths-III scales. There was a significant negative correlation of moderate strength between follow-up Phe levels and DQ of all Griffiths-III scales.

**Table 4:** Correlation between phenylalanine level and Griffiths-III developmental scales

	FU Phe in the last 1 year		FU Phe in the last 3 months	
Griffith subscales	*r*	*P*	*r*	*p*
subscaleA_DQ (mean ± SD)	− 0.498	**0.003**	− 0.624	**0.000**
subscaleB_DQ (mean ± SD)	− 0.449	**0.006**	− 0.572	**0.001**
subscaleC_DQ (mean ± SD)	− 0.529	**0.001**	− 0.552	**0.002**
subscaleD_DQ (mean ± SD)	− 0.484	**0.003**	− 0.520	**0.003**
subscaleE_DQ (mean ± SD)	− 0.5	**0.002**	− 0.536	**0.002**
general_DQ (mean ± SD)	− 0.524	**0.001**	− 0.556	**0.001**

The bold *P*-value reflects a significant difference *r *Pearson coefficient, *Phe* phenylalanine, *SD *standard deviation,  *DQ* developmentalquotient

Table 5 shows a statistically significant difference between PKU phenotypes as regard the mean of follow-up Phe level in the last year and in the last 3 months, being higher in classic PKU than moderate and mild PKU.

Also, there is a statistically significant difference regarding the developmental quotient mean of subscales A, D, E, and general development, being lower in classic PKU than moderate and mild PKU.

**Table 5:** Comparison between PKU phenotypes as regard to follow-up Phe levels and Griffiths-III developmental scales

	Mild PKU *N* = 11	Moderate PKU *N* = 6	Classic PKU *N* = 13	*P* value*
Follow-up Phe level in the last year (mean ± SD)	429.1 ± 242.7	925 ± 281.5	1160.8 ± 521.3	** < 0.001**
Follow-up Phe levels in the last 3 months (mean ± SD)	431 ± 270.2	950 ± 242.9	1148.5 ± 543.8	**0.001**
subscaleA_DQ (mean ± SD)	91 ± 13.2	77.2 ± 22	71.7 ± 17.9	**0.034**
subscaleB_DQ (mean ± SD)	90.2 ± 13.9	83.3 ± 17.9	78.5 ± 15.8	0.206
subscaleC_DQ (mean ± SD)	97.9 ± 12.3	91.2 ± 20.6	81.1 ± 18.5	0.068
subscaleD_DQ (mean ± SD)	108.7 ± 8.6	93.5 ± 21.4	90.2 ± 21.2	**0.045**
subscaleE_DQ (mean ± SD)	115.3 ± 8.9	100.2 ± 13.2	95.9 ± 11.9	**0.001**
general_DQ (mean ± SD)	100.6 ± 13.7	83.7 ± 24.5	78.7 ± 20.1	**0.027**

*One-way ANOVA test

The bold *P*-value reflects a significant difference

## Discussion

Precise treatment of PKU is a crucial step to prevent mental retardation. There is a lack of studies that have examined the behavioral and cognitive effects of metabolic control in individuals with PKU at various ages. Therefore, it is time to establish standardized PKU testing protocols for this rare disease, aiming to expand the treatment strategies beyond the traditional methods to achieve the better neurodevelopmental outcome for diseased children.

Our study included 30 PKU children, where 63.3% were from rural areas where positive consanguineous marriage is common; positive consanguinity was present in 73.3% of our children. A positive family history was found in 26.7% of cases, which includes characteristics of autosomal recessive inherited diseases were positive Consanguinity is a risk factor, and the recurrence of disease in the following sibling is 25% [17].

Our study was the first to assess the neurodevelopmental outcome of early treated children with PKU compared to healthy group using Griffiths-III developmental scales. We found a significant decrease in Griffiths-III DQ of general development among diseased children. The general development of Griffiths-III is described as the mean of five subscales of child development (foundation of learning, language and communication, eye and hand coordination, personal-social-emotional, and gross motor).

Of note, each subscale of Griffiths-III was tested individually, and we found a significant decrease in DQ of Griffiths-III foundation of learning subscale (subscale A) among diseased children. This subscale allows for a unique exploration of a child executive functioning and working memory as it assesses the child skills for learning, ways of thinking, memory, and problem-solving abilities [12].

This finding aligns with a meta-analysis conducted by DeRoche and Welsh which examined 25 years of neurocognitive outcomes in children and adolescents with PKU, spanning from 1980 to 2004 and including 33 studies. The effect sizes for intelligence, compared to unrelated controls, ranged from small to moderate (0.20 to 0.42). In contrast, the effect sizes for executive functioning and its component processes were in the “moderate to large” range. DeRoche and Welsh concluded that individuals who receive early and continuous treatment are likely to have IQ scores within the average range; however, they may still experience significant impairments in executive functioning [18].

Also, our study showed a significant decrease in DQ of Griffiths-III language and communication subscale (subscale B) among PKU children. This subscale assesses a child’s development of speech and language and the ability to use language to engage in social interaction with others. The subscale looks at expressive communication, receptive language development, communicative intent, and verbal memory [12].

This finding comes with a study performed among PKU children and control subjects in a pediatric hospital in Tehran which revealed that the language skills performance of control subjects was significantly better than that of early-treated subjects (*P* < 0.001) [19]. The findings related to spoken language suggest that this metabolic disorder affects language abilities even in individuals diagnosed early.

These results contrast with those of Michel et al. [20] and Ozanne et al. [21], who found no significant linguistic deficits in early-treated subjects. The divergence between these studies and the present one may stem from the different linguistic domains assessed. In this study, individuals with PKU were found to be particularly susceptible to difficulties in organizing language, rather than other aspects of language. This may be attributed to the specific nature of the tasks employed in the assessment, which involved memory and semantic clustering that are closely associated with executive functioning [22].

Moreover, in the current study there was a significant decrease in DQ of Griffiths-III eye and hand coordination subscale (subscale C) among PKU group. This subscale assesses a child fine motor skills such as bilateral coordination, motor planning, visuo-motor integration, graphomotor skills, speed, and strength of movement [12]. Our result matched with a study comparing 19 ETPKU patients with 13 healthy controls on a visuo-motor task revealed a slower response in the PKU group [23].

Additionally, children with PKU demonstrated a significant decrease of DQ of Griffiths-III personal-social-emotional subscale (subscale D). This subscale assesses the child growing independence and proficiency in daily activities by measuring self-help, self-awareness, and self-care skills. It also assesses social interaction, attention, emotional expression and regulation, and empathy [12].

This finding comes with previous study that compared the social-cognitive abilities of 95 PKU patients with 95 healthy controls [7]. This delay could be attributed to the negative effects of temporary increases in Phe levels, which may also lead to a reduction in dopamine levels. Furthermore, it has been suggested that fluctuations in Phe levels over time could serve as a strong predictor of various cognitive and social deficits, such as those in executive functions, in individuals with PKU [24].

Gross motor skills (subscale E) in PKU children were lower than in controls in our study, although not reaching a significant level. Similar finding was shown in a study done among 70 Iranian children with ETPKU and 100 healthy and normal children matched with the ETPKU group for age. ETPKU Iranian children had lower motor development [25]. Therefore, the motor function is not a clue for normal neurodevelopment. As in our study, most diseased children achieved an average range of gross motor functions while the remaining subscales were low.

Among PKU children, only 5 cases were controlled and 25 cases were uncontrolled. Several factors contribute to diet non-adherence: unpalatable medical formula, unavailability, high cost of protein substitutes, low parental education levels, and the low socioeconomic status for most cases (63.3% from rural areas).

Controlled and uncontrolled PKU children demonstrated a significant decrease in the Griffith mental subscales A, B, C, E, and general development among uncontrolled PKU children. This illustrates the negative impact of high phenylalanine levels.

This was also demonstrated in a study conducted among 56 PKU children in Brazil, which explored the causes and effects of diet non-adherence, thereby emphasizing the importance of the family as a factor that promotes treatment adherence [26].

In the current study, DQ of all Griffiths-III subscales had a significant negative correlation with Phe level follow-up in the previous year and FU level in the previous 3 months before assessment. This can be explained by insufficient Phe hydroxylase (PAH) activity which resulted in hypotyrosinemia and HPA [27]. High Phe reduces dopamine and serotonin levels in the brain by competing with tyrosine, tryptophan, and other large neutral amino acids (LNAAs) for the L-type amino acid carrier (LAT1) at the blood–brain barrier (BBB) [28]. Even when people are “on a diet” or have comparable plasma Phe levels, the transport of Phe across the BBB has been discovered to be quite varied, resulting in diverse clinical effects between individuals.

This copes with a previous study done among 27 Brazilian PKU children, which studied the impact of blood phenylalanine level variations on the development of executive functions and found that greater relative Phe-variation was linked to poorer executive functions [29].

The comparison of the follow-up level of phenylalanine between PKU phenotypes showed a significant difference, being higher in classic PKU than moderate and mild PKU. This means that the follow-up Phe level is higher when the initial Phe level is higher. It can predict a more challenging control of Phe levels and subsequently more complications and mental dysfunction in patients with elevated initial levels.

This explains the greater impairment of Griffith III mental scales in classic PKU than in moderate and mild PKU, being significantly different of the developmental quotient of subscales A,C, D, and general development. So, more dietary restriction and close monitoring are recommended for classic PKU children.

This confirms the negative impact of long-term uncontrolled Phe levels.

Hyperphenylalaninemia disrupts neurotransmission and cerebral protein synthesis through hypotyrosinemia, poor transport of big amino acids into the brain, and impairment of other enzymatic systems. PAH deficiency reduces tyrosine biosynthesis, and HPA leads to competitive binding at the LAT1 carrier. This results in Phe preferentially crossing the BBB and a lack of other LNAAs such as tryptophan and tyrosine, which are building blocks of serotonin and dopamine [30]. Therefore, the negative correlation between Phe levels and the neurodevelopmental outcome may highlight the importance of reevaluation of the Phe levels threshold in treatment decision making.

Every PKU participant in our study was diagnosed via neonatal screening and began dietary treatment promptly. However, the consistently high phenylalanine levels suggest a lack of adherence to the diet over the long term. This lack of control is likely a significant factor contributing to the deficits in the Griffiths-III mental subscales. In this regard, it is important to emphasize that although our study corroborates the neurodevelopmental risks in early-treated PKU children, these results are not representative of the strict metabolic adherent population. The stark contrasts observed between our PKU cohort and the control group were most likely influenced not only by the pathophysiology of PKU but also by the ongoing hyperphenylalaninemia due to inadequate treatment adherence.

Since none of the cases we looked at had any developmental delays, our results should be interpreted cautiously because it seems that children with PKU have trouble with mental functioning. This highlights the importance of including comprehensive assessments and ongoing monitoring of brain function, rather than solely emphasizing the evaluation of gross motor milestones. Also strict metabolic control is essential.

### Limitations and future directions

This study was a retrospective observational study conducted at a single center with a limited number of participants. Each patient was assessed only once, and at different ages, limiting the ability to observe developmental progress over time. The retrospective nature of the study also prevented the inclusion of baseline neurodevelopmental evaluations and hindered the ability to establish consistent correlations between longitudinal phenylalanine levels and neurodevelopmental outcomes. Additionally, the absence of neuroimaging, genetic testing, and comprehensive cognitive assessment tools restricted a thorough evaluation of the potential neurotoxic effects of elevated phenylalanine levels on the developing brain.

Considering these limitations, there is a strong recommendation to develop programs for early detection and intervention targeting neurodevelopmental challenges in children with PKU. It is equally important to provide counseling for parents, educators, and affected individuals to raise awareness of possible academic and social difficulties. Educational interventions that enhance self-regulation and cognitive control are crucial for promoting academic achievement and social adaptation.

Future studies with a longitudinal follow-up design are needed to offer deeper insights into the cognitive and neurodevelopmental trajectories of ETPKU children. Such research could help identify subtle or progressive changes in mental functioning, ultimately contributing to a more comprehensive understanding of long-term outcomes in this population.

## Conclusion

Children with PKU who receive early treatment remain at increased risk for impairments in cognitive, language, social, and fine motor skills, despite having normal gross motor development, when compared to typically developing children. These deficits are negatively correlated with phenylalanine levels, indicating that higher phenylalanine concentrations are associated with poorer neurodevelopmental outcomes.

## Data Availability

No datasets were generated or analysed during the current study.

## Abbreviations

BBB: Blood brain barrier
BH4: Tetrahydrobiopterin
ETPKU: Early treated PKU
HPA: Hyperphenylalaninemia
LAT1: L-type amino acid carrier
LNAAs: Large neutral amino acids
NBS: Newborn screening
PAH: Phenylalanine hydroxylase
Phe: Phenylalanine
PKU: Phenylketonuria
